# Supplementary figures and images for: Evidence for hybrid breakdown in production of red carotenoids in the marine invertebrate Tigriopus californicus
Source: PLoS One. 2021 Nov 8;16(11):e0259371. doi: 10.1371/journal.pone.0259371 (PMC8575244; doi:10.1371/journal.pone.0259371)

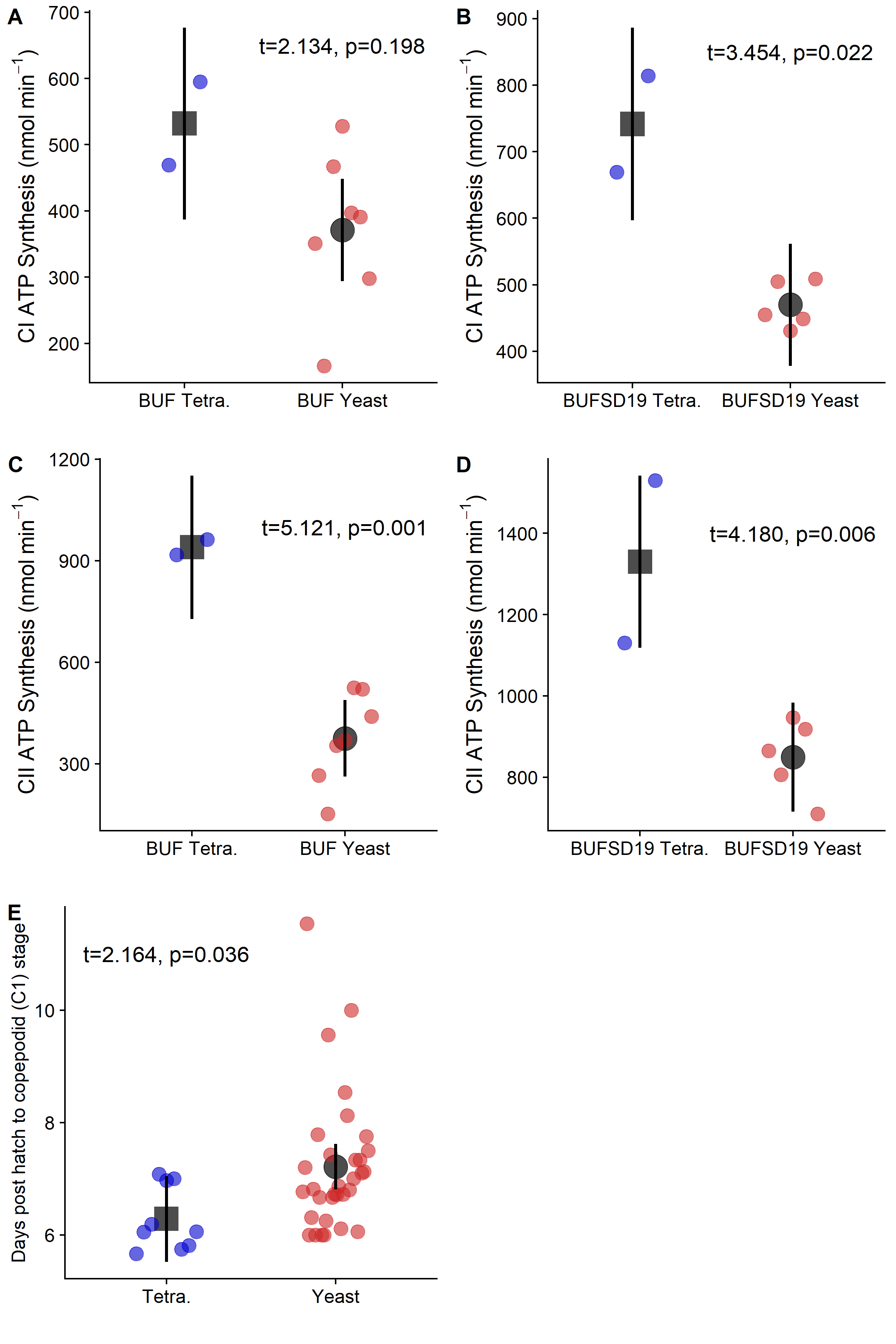

Supplement: S1 Fig — Individual replicates from yeast-fed copepods are shown in red and data from algae-fed copepods are in blue. A) Difference in Complex I ATP production between the La Bufadora parental line on each diet, B) difference in Complex I ATP production between hybrid copepods from the BUF x SD 19 line on each diet, C) difference in Complex II ATP production from the La Bufadora parental line on each diet, D) difference in Complex II ATP production between the hybrid copepods from the BUF x SD 19 line on each diet, and E) difference in offspring development time between all parental copepod lines fed either algae or yeast. Black dots are group averages and black bars are 95% confidence intervals around the mean. (TIF) [file pone.0259371.s001.tif]

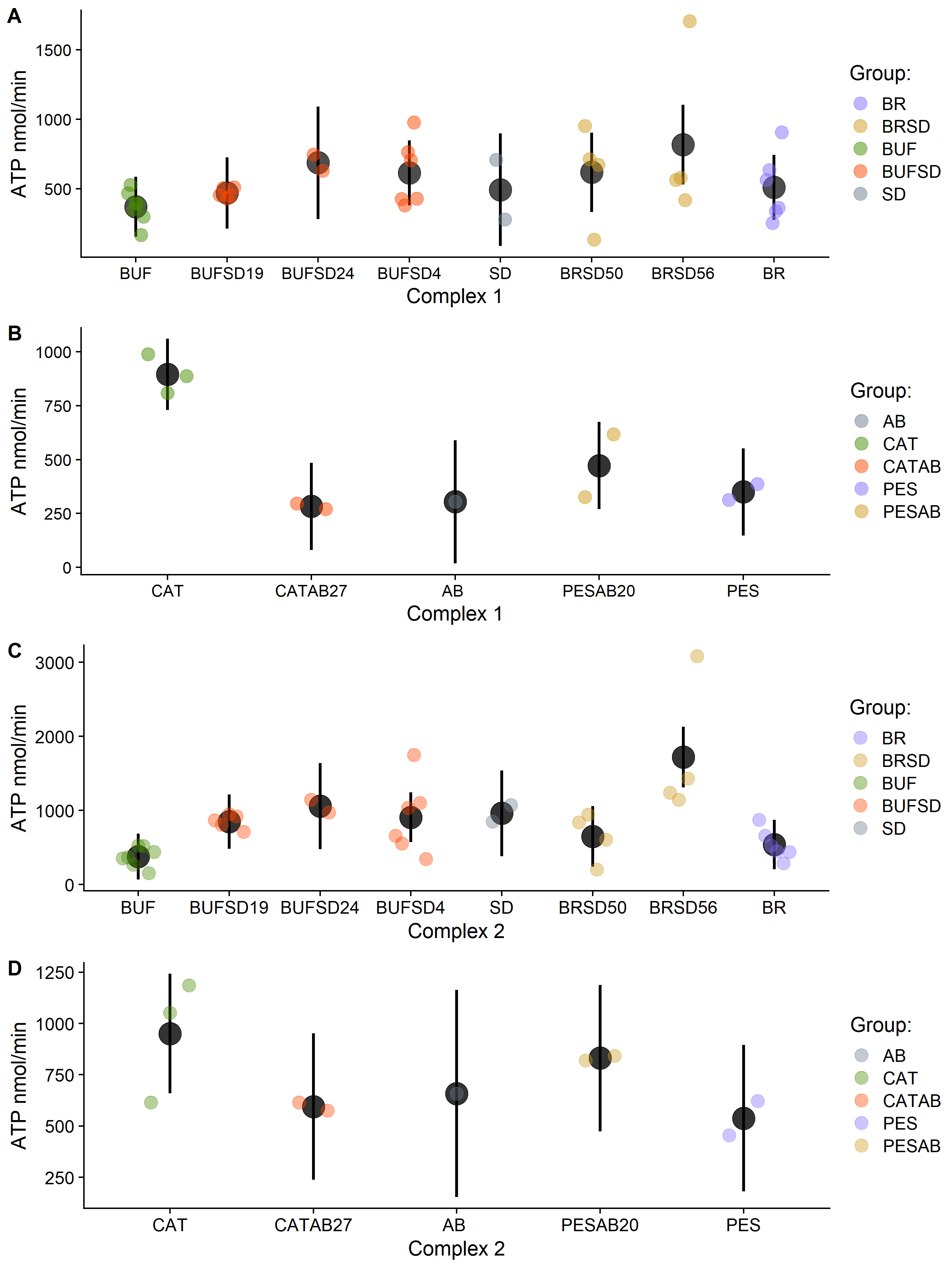

Supplement: S2 Fig — Differences among hybrid RILs and non-hybrid PILs from multigeneration crosses fed yeast only in A-B) Complex I ATP production and C-D) Complex II ATP production. Colored dots represent replicates within each line that are the average ATP produced by isolated mitochondria from 20 pooled copepods. Dark grey circles are the line average. Black lines and colored shading are 95% confidence intervals around the line average. Where 95% confidence intervals do not overlap, there is a statistically significant difference between lines. (TIF) [file pone.0259371.s002.tif]

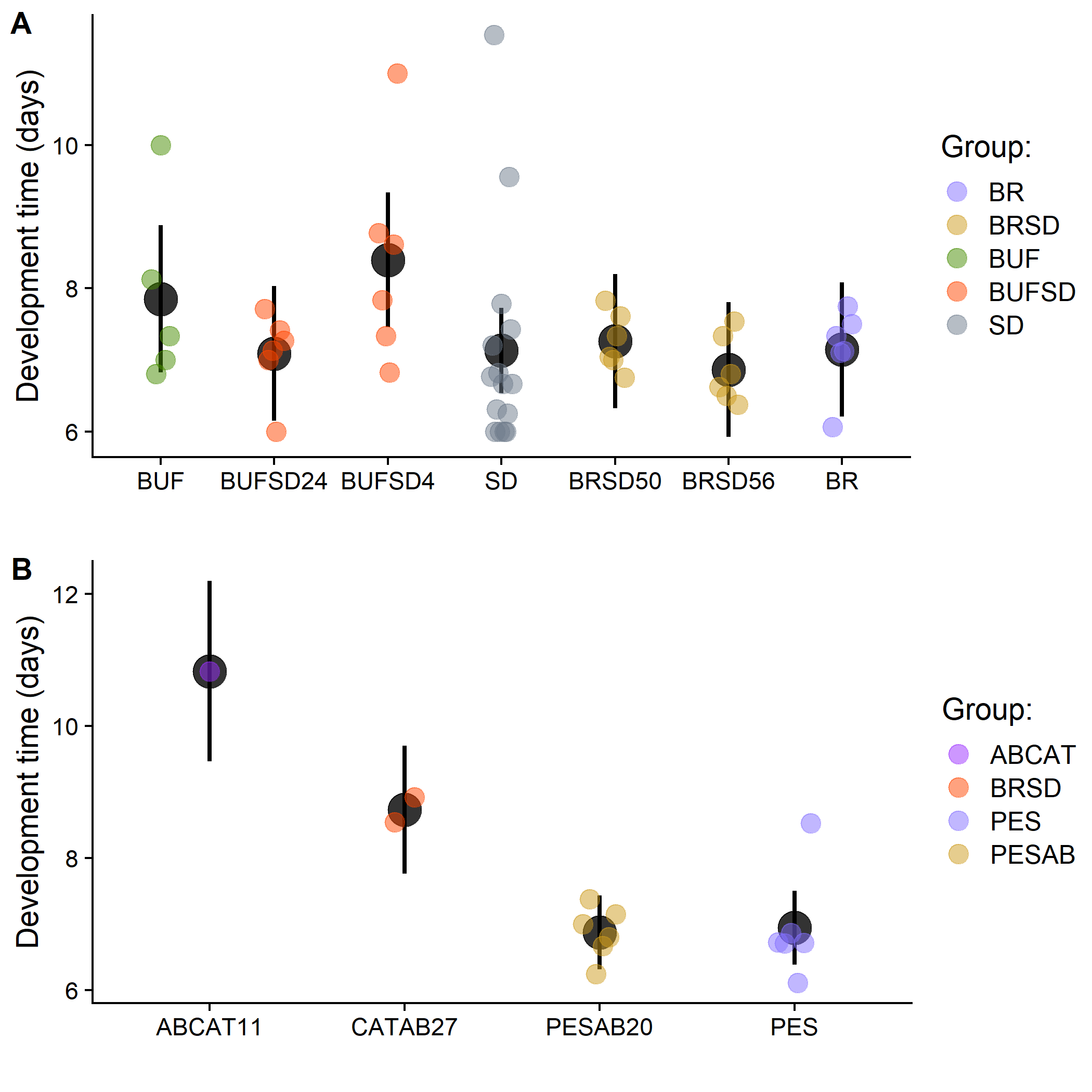

Supplement: S3 Fig — Colored dots represent replicates within each line that are the average values produced by individual females. Dark grey circles are the line average. Black lines and colored shading are 95% confidence intervals around the line average. Where 95% confidence intervals do not overlap, there is a statistically significant difference between lines. (TIF) [file pone.0259371.s003.tif]

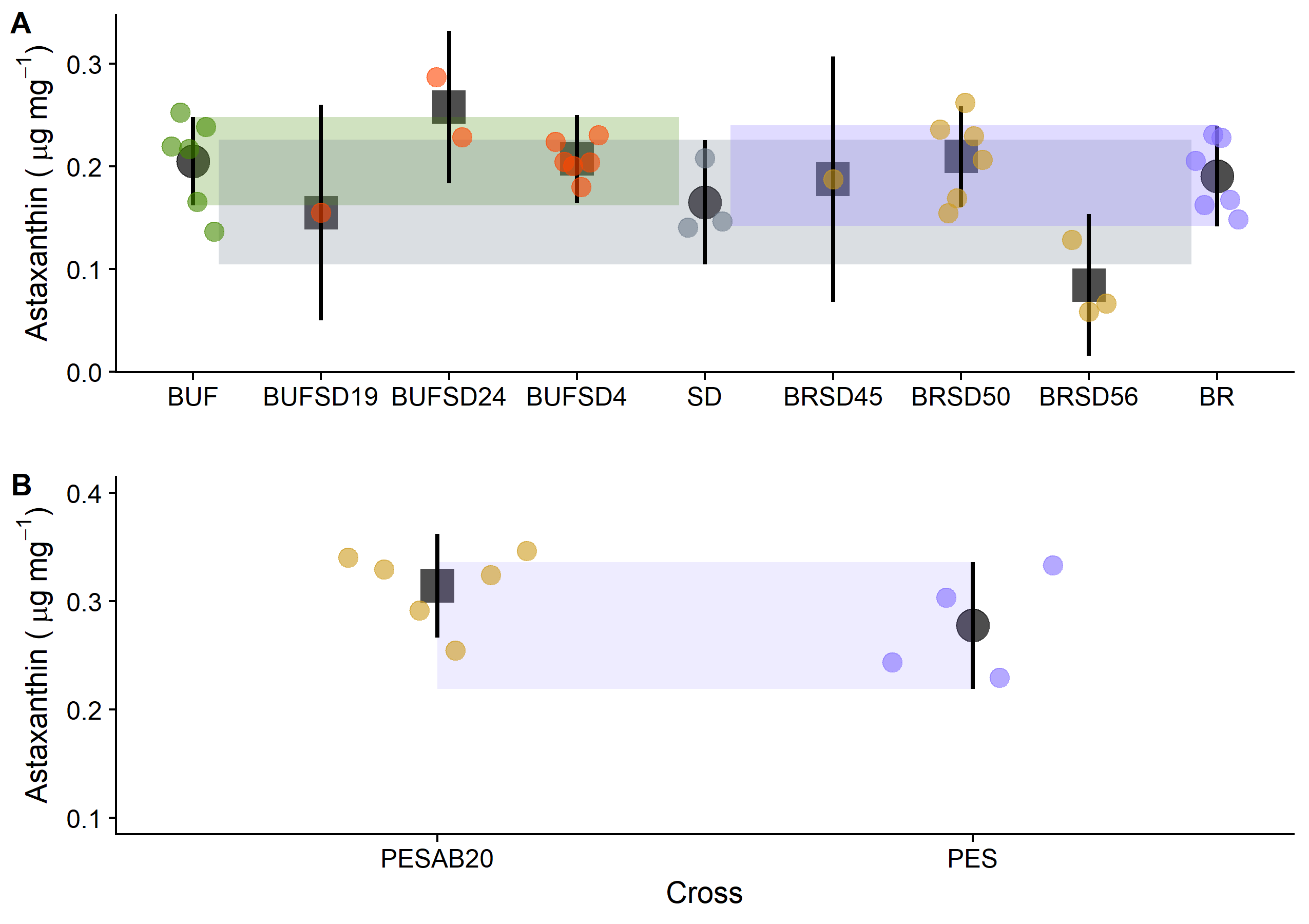

Supplement: S4 Fig — Colored dots represent replicates within each line that are the average astaxanthin level of approximately 10 copepods. Dark grey circles (parental lines) and dark grey squares (hybrid lines) are the line average. Black lines and colored shading are 95% confidence intervals around the line average. Where 95% confidence intervals do not overlap, there is a statistically significant difference between lines. (TIF) [file pone.0259371.s004.tif]

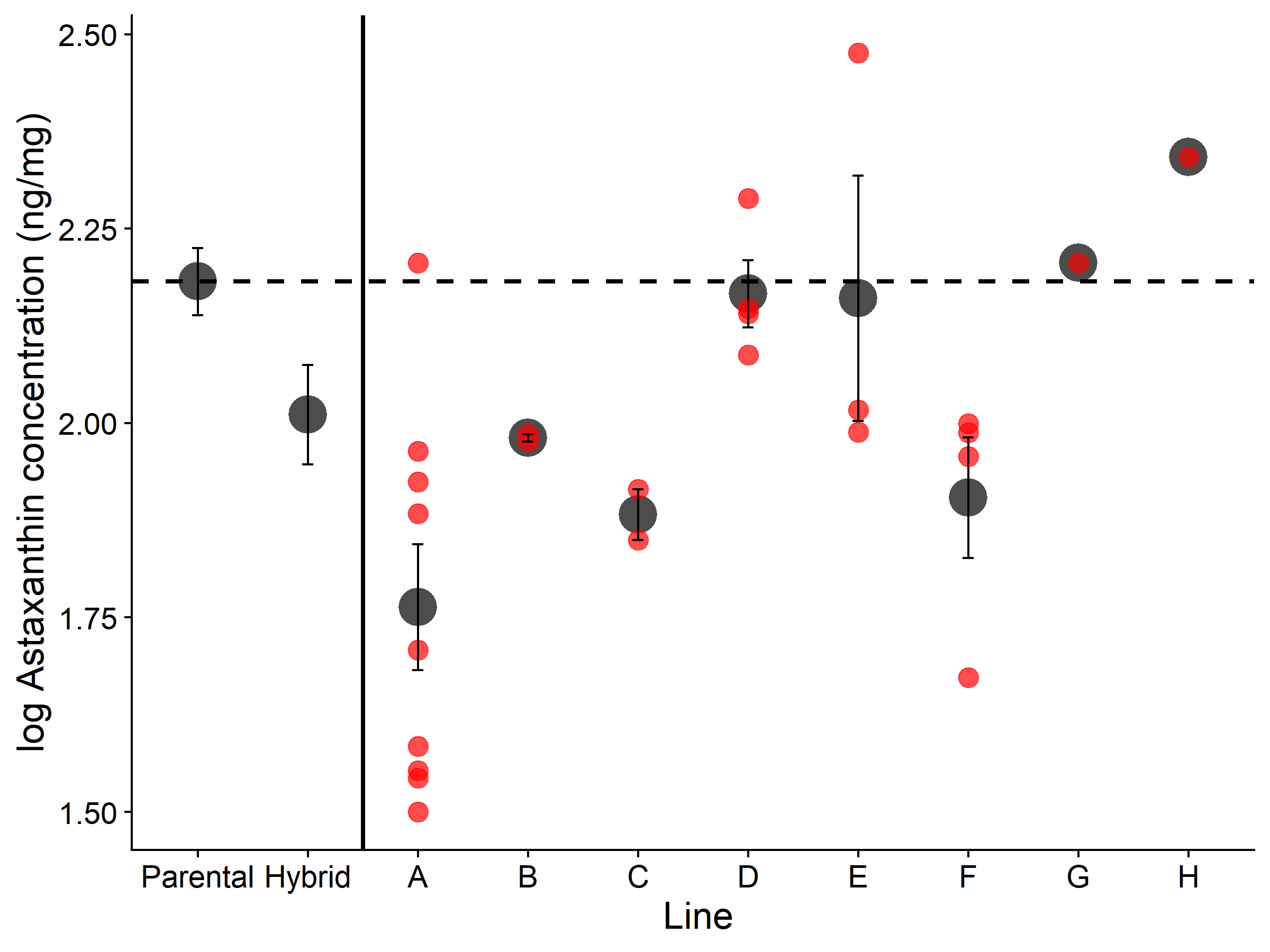

Supplement: S5 Fig — Line averages (black dots) and individual replicates (red dots) in astaxanthin production among AB x SD hybrid lines (A-H; ‘Hybrid’ represents average among all lines) and in parental (AB) copepods. Black bars show standard error. After one generation of inbreeding, 9 AB parental lines and 8 AB x SD hybrid lines were viable, while SD parental lines and SD x AB hybrid lines did not survive to carotenoid analysis. Previously, T. californicus has been shown to be highly sensitive to inbreeding [115], which is likely the cause of the mortality that we observed in SD parental and SD x AB hybrid lines. (TIF) [file pone.0259371.s005.tif]

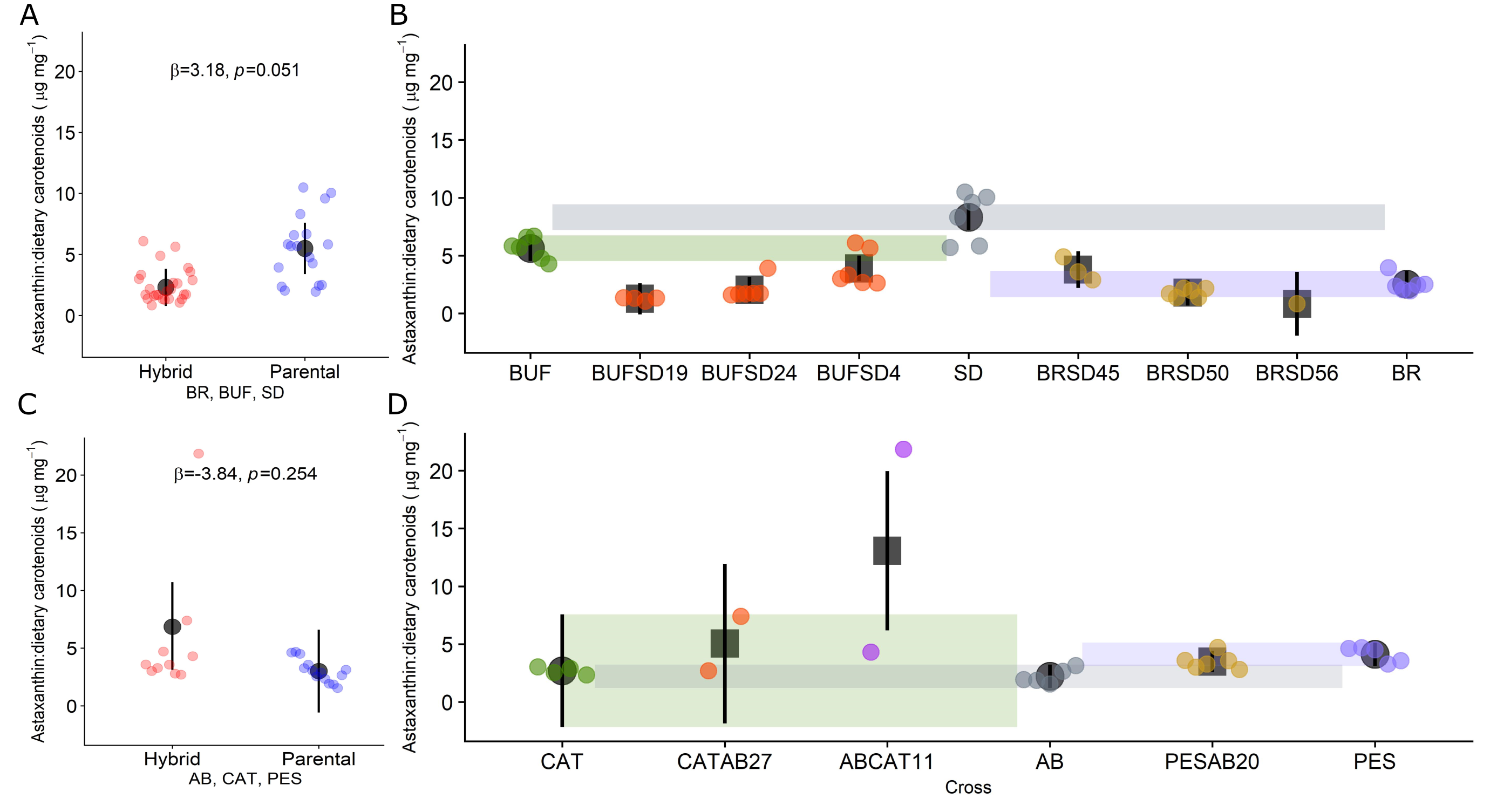

Supplement: S6 Fig — Colored dots represent replicates within each line that are the average carotenoid ratio of approximately 10 copepods. Dark grey circles (parental lines) and dark grey squares (hybrid lines) are the line average. Black lines and colored shading are 95% confidence intervals around the line average. Where 95% confidence intervals do not overlap, there is a statistically significant difference between lines (S4 Table). (TIF) [file pone.0259371.s006.tif]

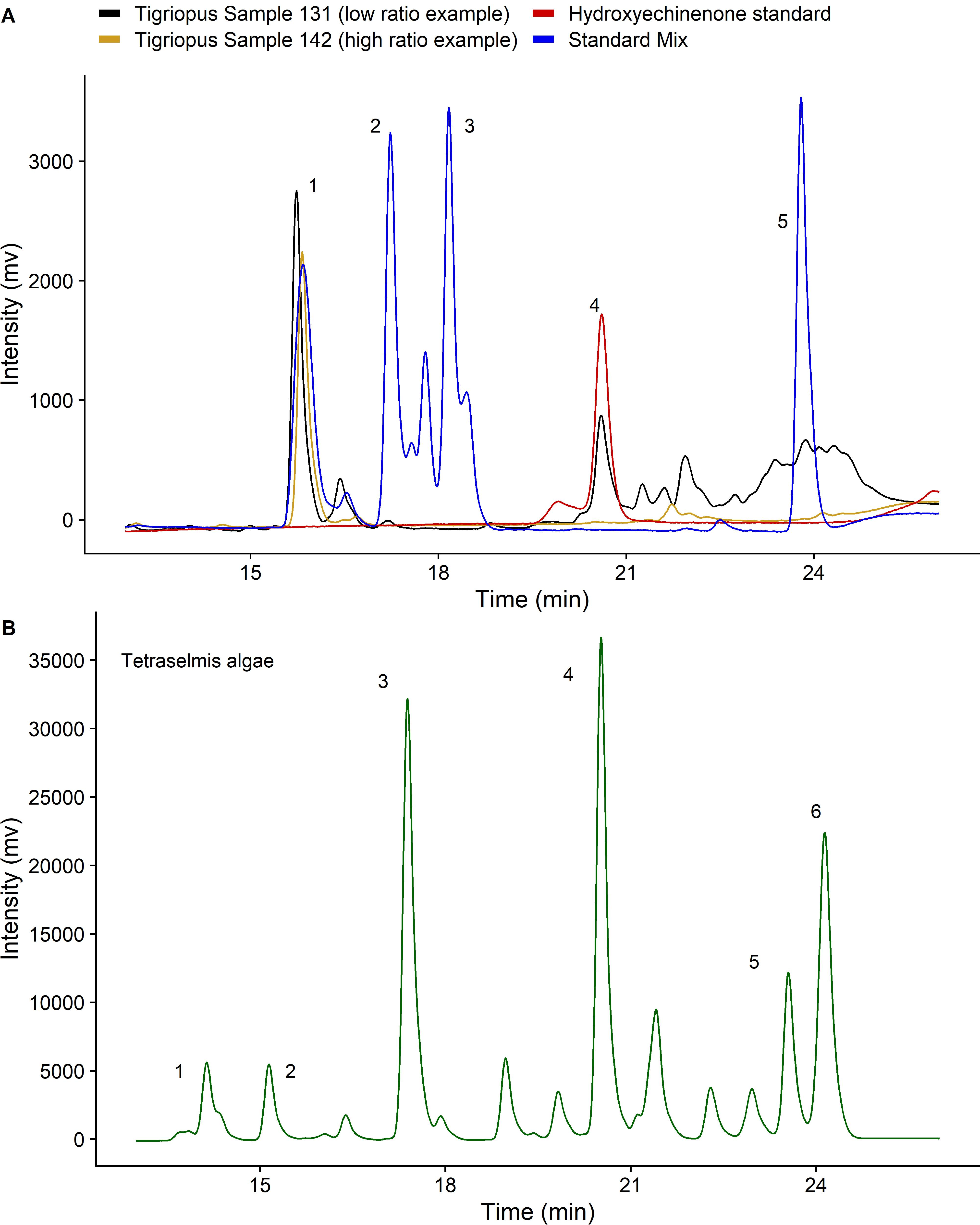

Supplement: S7 Fig — A) HPLC chromatograms of a mix of carotenoid standards (blue), hydroxyechinenone standard (red), a copepod sample with a low ratio of astaxanthin to dietary carotenoids (black), and a copepod samples with a high ratio of astaxanthin to dietary carotenoids (yellow). Numbered peaks are as follows: 1) astaxanthin, 2) lutein, 3) canthaxanthin, 4) hydroxyechinenone, 5) β-carotene. B) HPLC chroma profile of the Tetraselmis algae we fed the copepods during the experiment. Numbered peaks are as follows: 1) violaxanthin isomer, 2) trans-violaxanthin, 3) lutein, 4) hydroxyechinenone, 5) α-carotene, 6) β-carotene. Of these carotenoids, only trans-violaxanthin, hydroxyechinenone and β-carotene are precursors to astaxanthin [9, 32]. (TIF) [file pone.0259371.s007.tif]

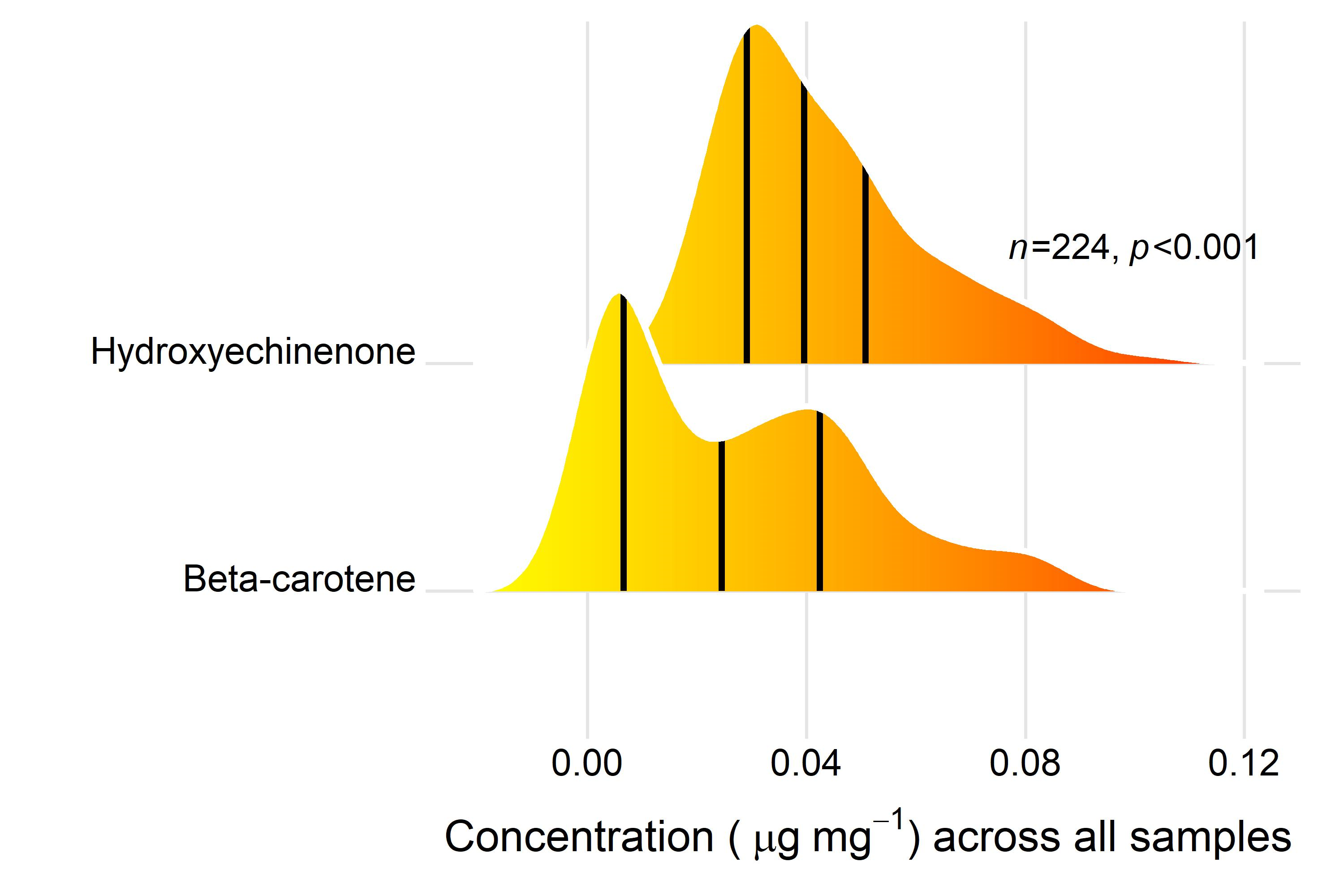

Supplement: S8 Fig — The black lines represent the median and upper/lower quartiles of each group. The p-value was derived from a linear mixed effects model with inbred line ID encoded as a random effect. (TIF) [file pone.0259371.s008.tif]

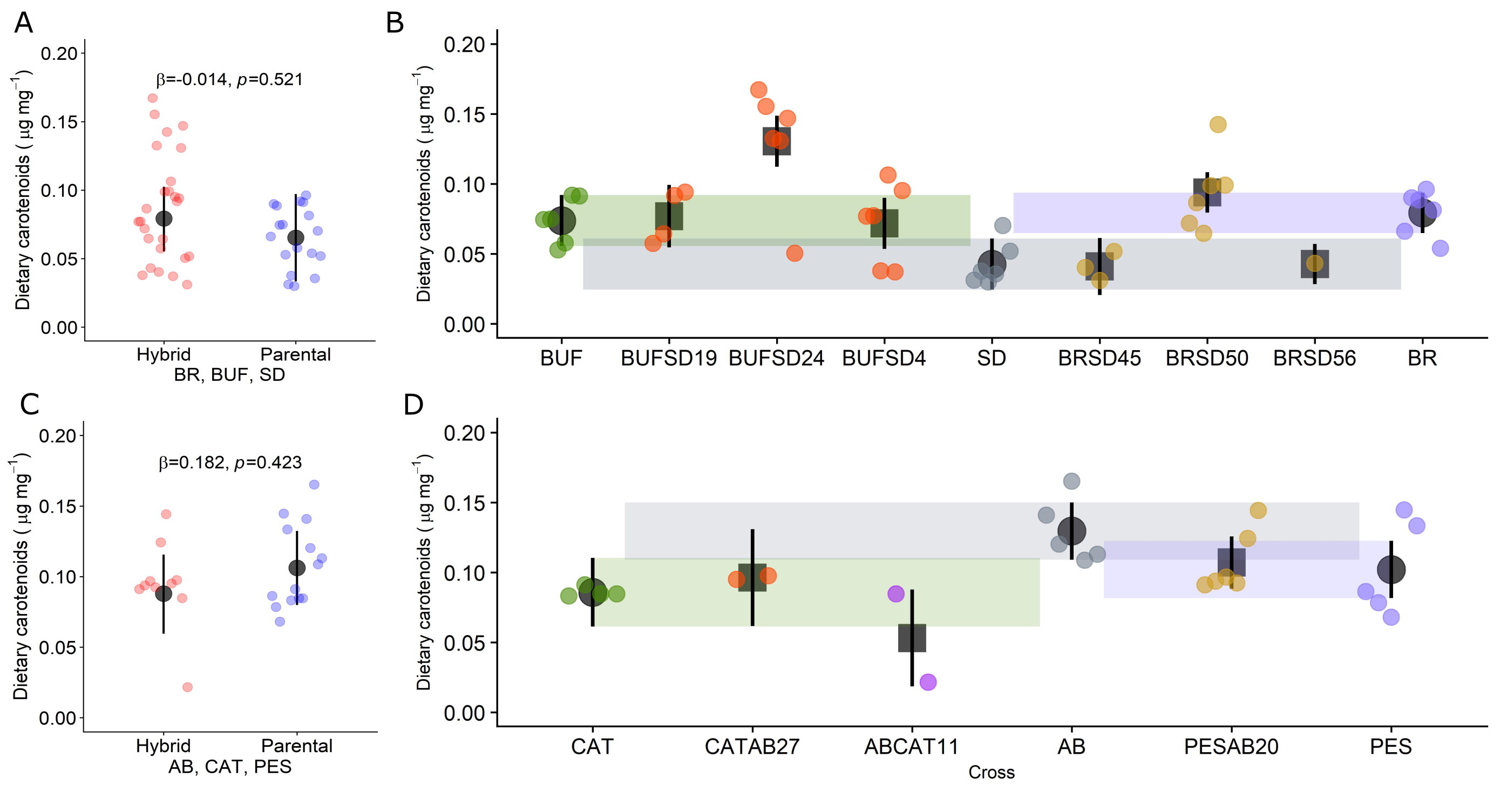

Supplement: S9 Fig — Colored dots represent replicates within each line that are the average of approximately 10 copepods. Dark grey circles (parental lines) and dark grey squares (hybrid lines) are the line average. Black lines and colored shading are 95% confidence intervals around the line average. Where 95% confidence intervals do not overlap, there is a statistically significant difference between lines (S5 Table). (TIF) [file pone.0259371.s009.tif]

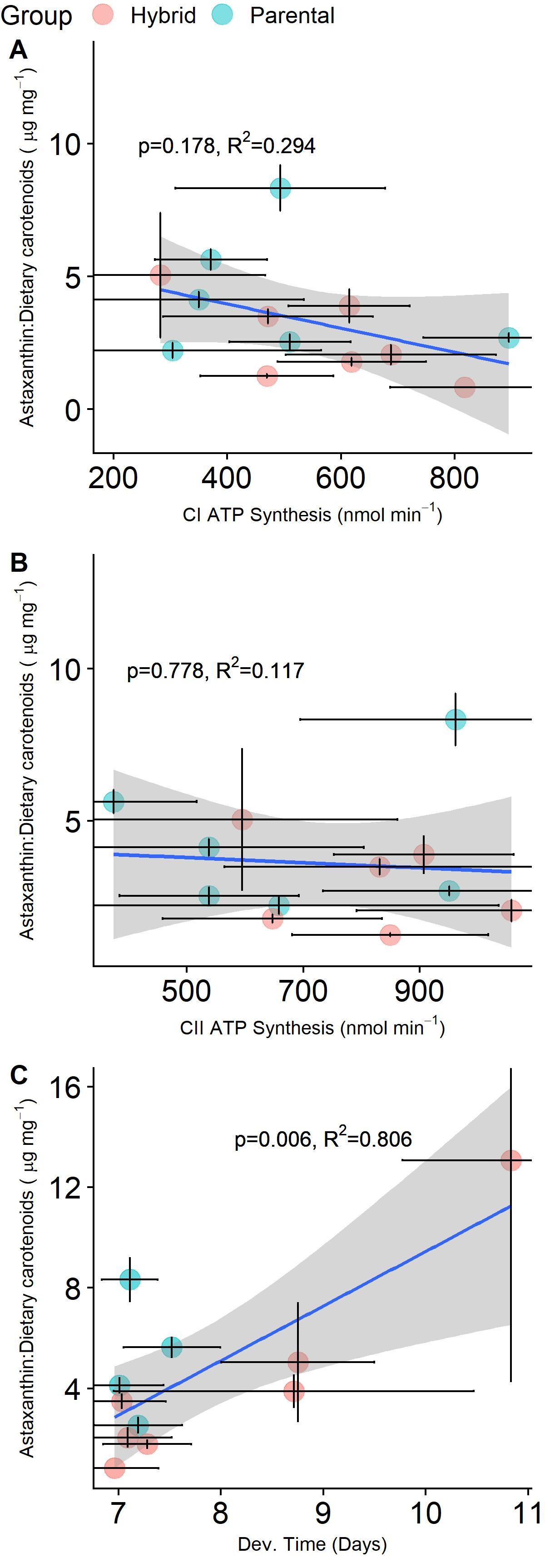

Supplement: S10 Fig — Colored dots represent line averages and the vertical and horizontal black bars extending from the colored dots represent the standard error of each trait. The grey shading is the 95% confidence interval around the model estimated slope. Adjusted R2 values are shown. (TIF) [file pone.0259371.s010.tif]

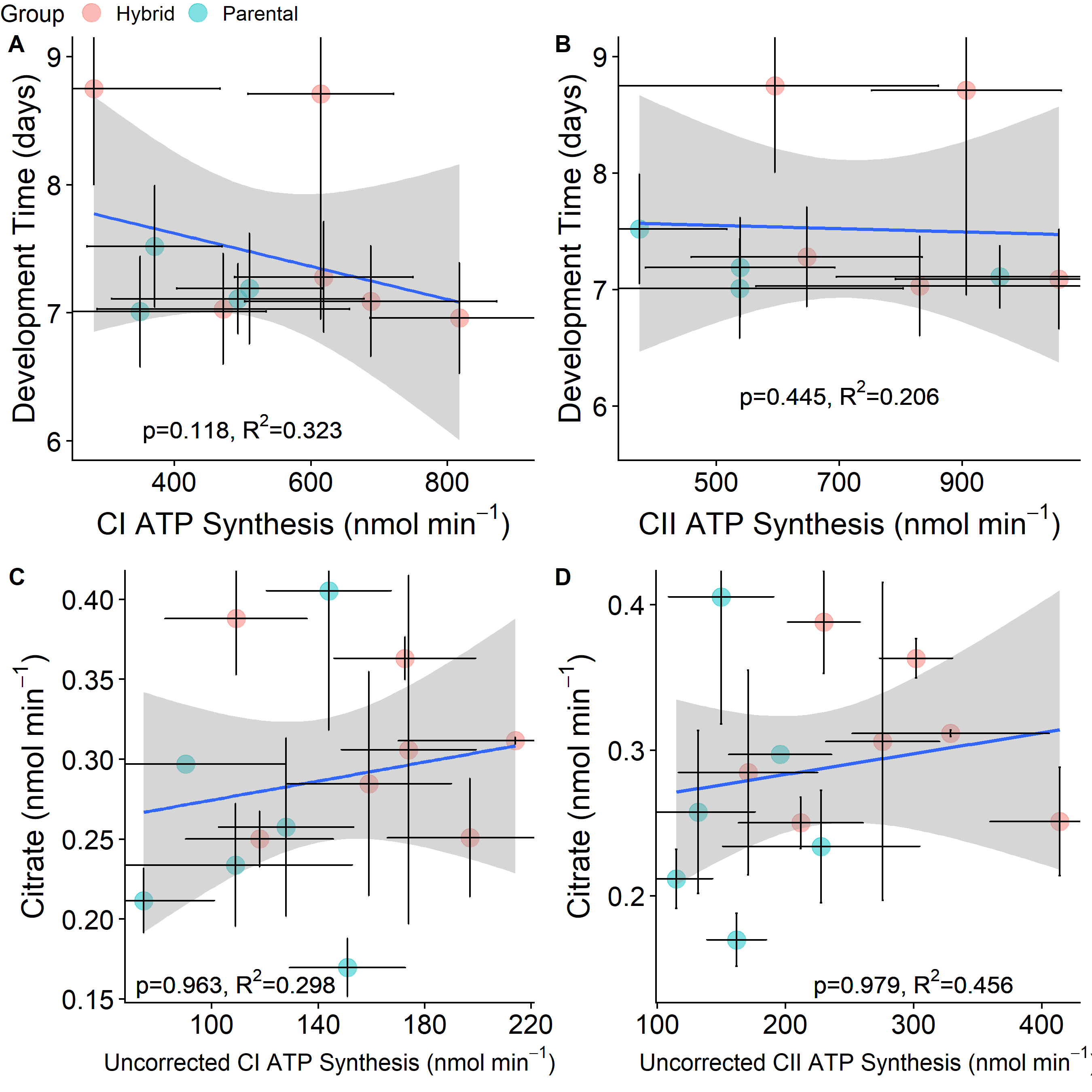

Supplement: S11 Fig — Relationships between ATP production and either offspring development time (days post hatch to first copepodid stage; A and B) or mitochondrial volume (C and D) among hybrid and non-hybrid lines. Colored dots represent line averages and the vertical and horizontal black bars extending from the colored dots represent the standard error. The grey shading is the 95% confidence interval around the model estimated slope. Adjusted R2 values are shown. (TIF) [file pone.0259371.s011.tif]

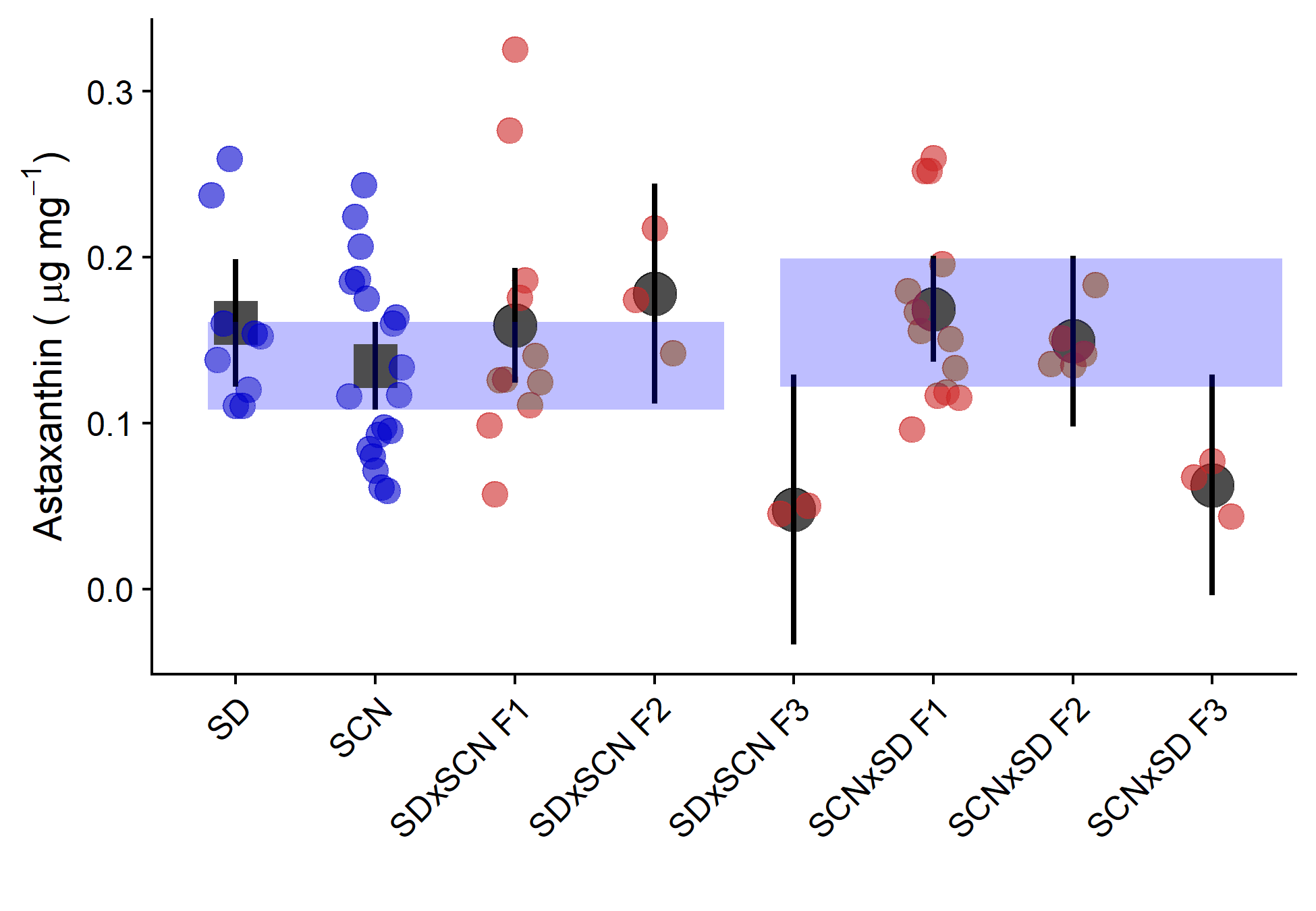

Supplement: S12 Fig — Groups are split by the direction of the cross (SCN female x SD male on the left, and SD female x SCN male on the right). Colored dots represent replicates within each group that are the average astaxanthin level of approximately 10 copepods per replicate. Dark grey circles are the group average. Black lines and colored shading are 95% confidence intervals around the group average. Where confidence intervals do not overlap, there is a statistically significant difference between groups. (TIF) [file pone.0259371.s012.tif]

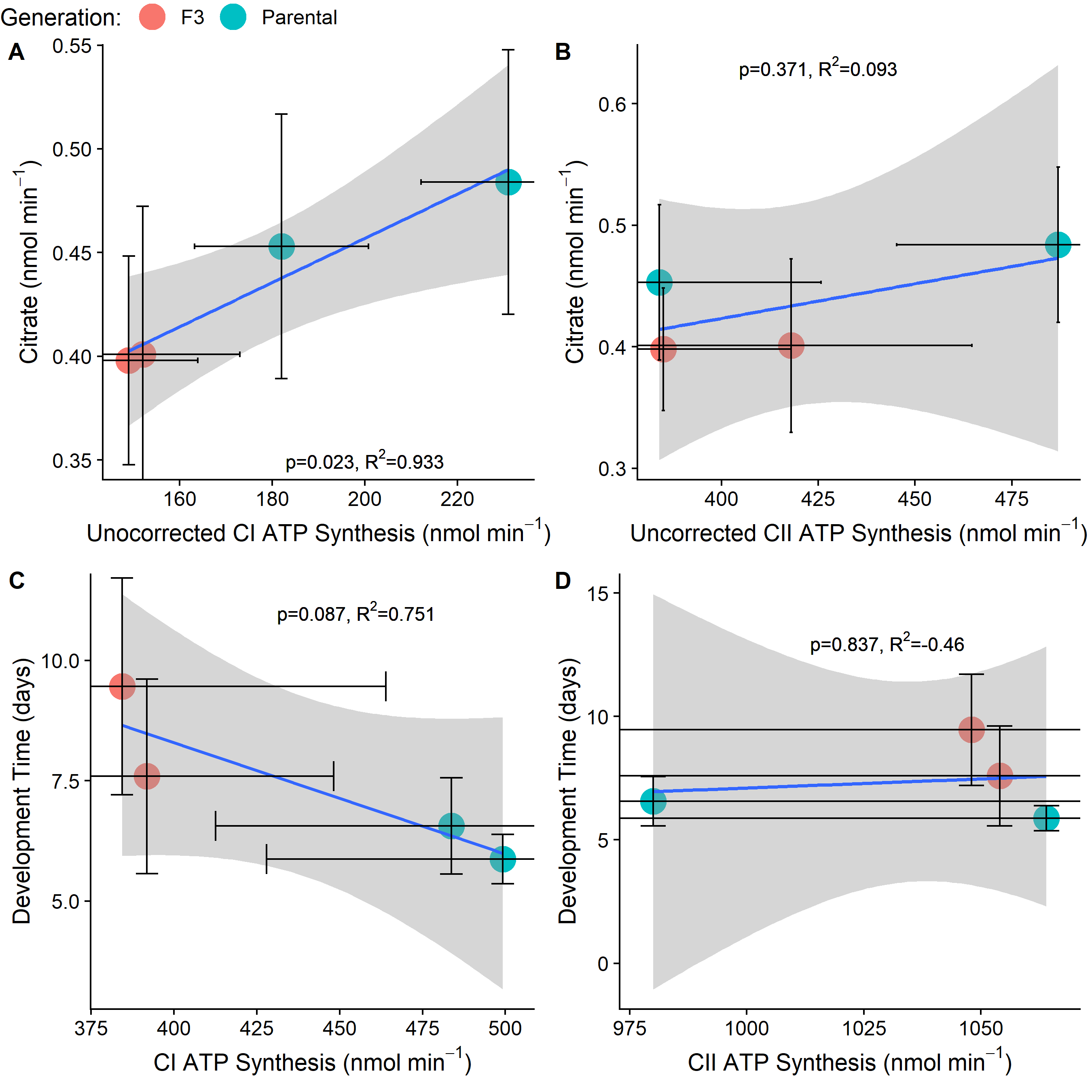

Supplement: S13 Fig — Relationships between ATP production and mitochondrial volume (A and B) or offspring development time (C and D) among hybrid and non-hybrid lines from the reciprocal cross between SD and SCN populations. Colored dots represent line averages and the vertical and horizontal black bars extending from the colored dots represent the standard error. The grey shading is the 95% confidence interval around the model estimated slope. Adjusted R2 values are shown. (TIF) [file pone.0259371.s013.tif]

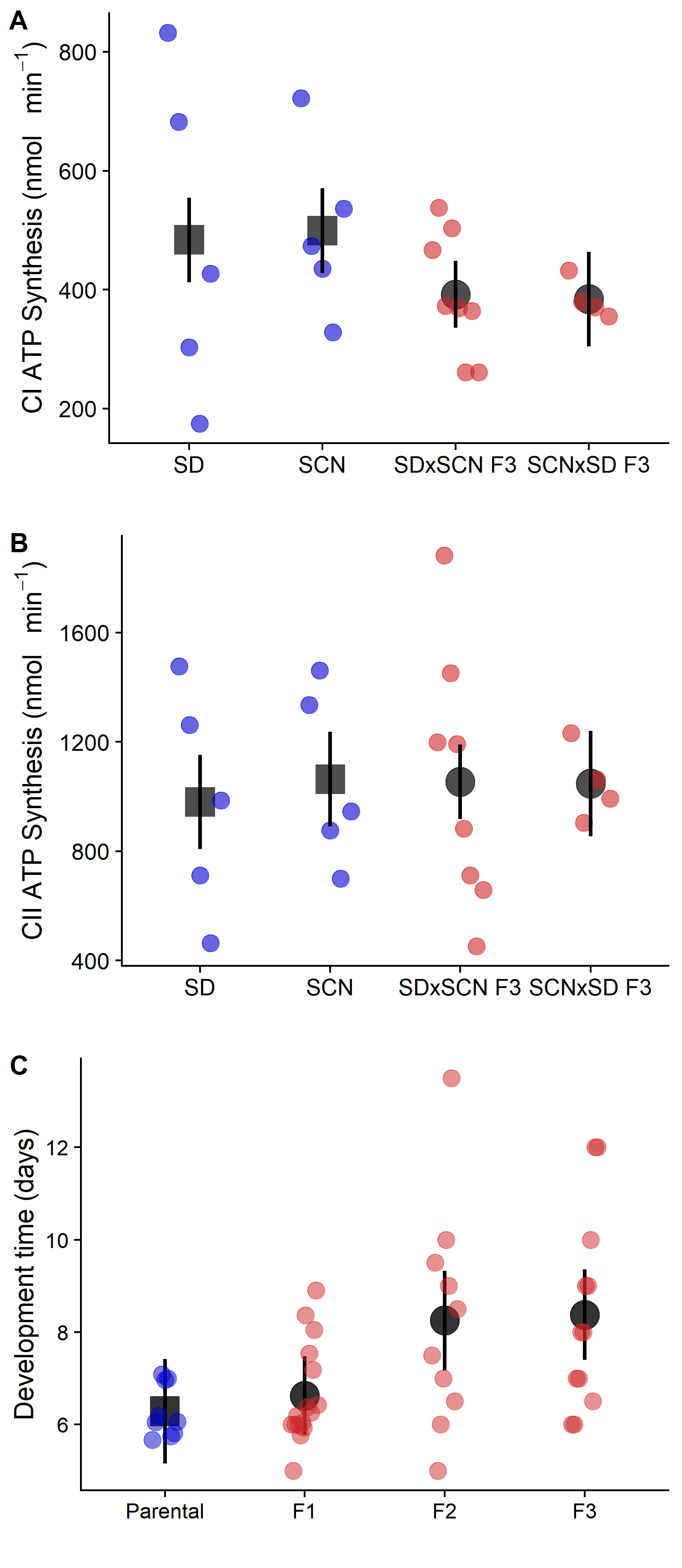

Supplement: S14 Fig — Differences among hybrid RILs and non-hybrid PILs from the reciprocal cross in A) Complex I ATP production, B) Complex II ATP production, and C) offspring development rate. Colored dots represent replicates within each line that are either the average ATP produced by isolated mitochondria from 20 pooled copepods (A and B) or clutch averages from individual females (C). Large, dark grey circles and squares are the line average. Black lines are 95% confidence intervals around the line average. Where 95% confidence intervals do not overlap, there is a statistically significant difference between lines. (TIF) [file pone.0259371.s014.tif]
