# Supplementary material for: Evidence for hybrid breakdown in production of red carotenoids in the marine invertebrate Tigriopus californicus
Source: PLoS One. 2021 Nov 8;16(11):e0259371. doi: 10.1371/journal.pone.0259371 (PMC8575244; doi:10.1371/journal.pone.0259371)
Supplement: S1 Table — The number of the replicates per line for each measurement and the approximate number of individual copepods per replicate. Shown as: # of line replicates (approx. # of individuals per replicate). (DOCX) [file pone.0259371.s015.docx]

| **S1 Table. A list of the lines from the experimental crosses used in this study.** The number of the replicates per line for each measurement and the approximate number of individual copepods per replicate. Shown as: # of line replicates (approx. # of individuals per replicate) | | | | |
| --- | --- | --- | --- | --- |
| **Line**  **(Female pop. x Male pop.)** | **Astaxanthin** | **Offspring development rate** | **ATP** | **Citrate synthase** |
| **SD x AB early generation RILs** | | | | |
| AB x AB | 9 (10) | - | - | - |
| AB x SD | 8 (10) | - | - | - |
| **SD-centric and AB-centric multigeneration RILs** | | | | |
| SD x SD | 6 (14) | 15 (15) | 2 (20) | 2 (20) |
| BUF x BUF | 7 (14) | 5 (6) | 7 (20) | 7 (20) |
| BR x BR | 7 (16) | 6 (15) | 6 (20) | 6 (20) |
| AB x AB | 5 (16) | - | 1 (20) | 1 (20) |
| CAT x CAT | 4 (15) | - | 3 (20) | 3 (20) |
| PES x PES | 6 (15) | 6 (13) | 2 (20) | 2 (20) |
| BR x SD 45 | 3 (12) | - | - | - |
| BR x SD 50 | 7 (14) | 6 (21) | 4 (50) | 4 (50) |
| BR x SD 56 | 1 (10) | 6 (8) | 4 (56) | 4 (56) |
| BUF x SD 4 | 7 (14) | 6 (16) | 6 (20) | 6 (20) |
| BUF x SD 19 | 4 (15) | - | 5 (20) | 5 (20) |
| BUF x SD 24 | 6 (13) | 6 (12) | 2 (20) | 2 (20) |
| AB x CAT 11 | 2 (8) | 1 (6) | - | - |
| CAT x AB 27 | 2 (9) | 2 (12) | 2 (20) | 2 (20) |
| PES x AB 20 | 7 (14) | 6 (10) | 2 (20) | 2 (20) |
| **Reciprocal cross between SD and SCN** | | | | |
| SD parental | 9 (10) | 7 (20) | 5 (20) | 4 (17) |
| SCN parental | 19 (10) | 8 (20) | 5 (20) | 5 (11) |
| F1 generation | 24 (10) | - | - | 16 (10) |
| F2 generation | 8 (10) | - | - | 10 (5) |
| F3 generation | 5 (10) | 12 (20) | 12 (20) | 12 (6) |
