# Supplementary material for: Evidence for hybrid breakdown in production of red carotenoids in the marine invertebrate Tigriopus californicus
Source: PLoS One. 2021 Nov 8;16(11):e0259371. doi: 10.1371/journal.pone.0259371 (PMC8575244; doi:10.1371/journal.pone.0259371)
Supplement: S3 Table — The model beta estimate represents the difference in the means per group. The confidence limits in the right two columns represent the confidence boundaries around the model estimate. (DOCX) [file pone.0259371.s017.docx]

| **S3 Table. Results from statistical models of paired contrasts of astaxanthin production among copepods from preliminary RILs from the cross between SD and AB populations.** The model beta estimate represents the difference in the means per group. The confidence limits in the right two columns represent the confidence boundaries around the model estimate. | | | | | | |
| --- | --- | --- | --- | --- | --- | --- |
| **Reference group** | **Contrast** | **Model estimate (*β_1_*)** | **SE** | ***P*-value** | **Lower 95% CL** | **Upper 95% CL** |
| **Hybrid vs non-hybrid averages** | | | | | | |
| Non-hybrids | Hybrids | -0.169 | 0.077 | 0.0444 | -0.320 | -0.018 |
| **Parental lines vs individual RILs (letters match figure S…)** | | | | | | |
| AB x AB | A | -0.417 | 0.085 | <0.0001 | -0.591 | -0.243 |
|  | B | -0.199 | 0.140 | 0.1675 | -0.487 | 0.089 |
|  | C | -0.298 | 0.140 | 0.0434 | -0.586 | -0.009 |
|  | D | -0.014 | 0.108 | 0.8994 | -0.235 | 0.208 |
|  | E | -0.020 | 0.120 | 0.8717 | -0.265 | 0.226 |
|  | F | -0.276 | 0.108 | 0.0167 | -0.498 | -0.054 |
|  | G | 0.026 | 0.189 | 0.8910 | -0.363 | 0.415 |
|  | H | 0.162 | 0.189 | 0.3996 | -0.227 | 0.551 |
