# Supplementary material for: Evidence for hybrid breakdown in production of red carotenoids in the marine invertebrate Tigriopus californicus
Source: PLoS One. 2021 Nov 8;16(11):e0259371. doi: 10.1371/journal.pone.0259371 (PMC8575244; doi:10.1371/journal.pone.0259371)
Supplement: S4 Table — The model beta estimate represents the difference in the means per group. The confidence limits in the right two columns represent the confidence boundaries around the model estimate. Bold lines denote a significant difference between the contrast and reference groups. (DOCX) [file pone.0259371.s018.docx]

| **S4 Table.** **Results from statistical models of paired contrasts of astaxanthin: dietary carotenoids among male copepods from multigeneration RILs and PILs.** The model beta estimate represents the difference in the means per group. The confidence limits in the right two columns represent the confidence boundaries around the model estimate. **Bold** lines denote a significant difference between the contrast and reference groups. | | | | | | |
| --- | --- | --- | --- | --- | --- | --- |
| **Reference group** | **Contrast** | **Model estimate (*β_1_*)** | **SE** | ***P*-value** | **Lower 95% CL** | **Upper 95% CL** |
| **Southern RILs and PILs** | | | | | | |
| BUF | **BUFSD19** | **4.375** | **0.859** | **0.0001** | **1.910** | **6.841** |
|  | **BUFSD24** | **3.582** | **0.768** | **0.0004** | **1.377** | **5.788** |
|  | BUFSD4 | 1.741 | 0.768 | 0.1796 | -0.465 | 3.946 |
| SD | **BUFSD19** | **7.066** | **0.859** | **<0.0001** | **4.600** | **9.531** |
|  | **BUFSD24** | **6.273** | **0.768** | **<0.0001** | **4.068** | **8.478** |
|  | **BUFSD4** | **4.431** | **0.768** | **<0.0001** | **2.226** | **6.636** |
|  | **BRSD45** | **4.536** | **0.957** | **0.0004** | **1.764** | **7.308** |
|  | **BRSD50** | **6.535** | **0.782** | **<0.0001** | **4.271** | **8.798** |
|  | **BRSD56** | **7.495** | **1.463** | **0.0001** | **3.262** | **11.729** |
| BR | BRSD45 | -1.247 | 0.957 | 0.6916 | -4.019 | 1.524 |
|  | BRSD50 | 0.751 | 0.782 | 0.8702 | -1.512 | 3.015 |
|  | BRSD56 | 1.712 | 1.463 | 0.7676 | -2.522 | 5.946 |
| **Northern RILs and PILs** | | | | | | |
| CAT | CATAB27 | -2.35 | 3.73 | 0.9202 | -13.995 | 9.30 |
|  | ABCAT11 | -10.37 | 3.73 | 0.0837 | -22.02 | 1.28 |
| AB | CATAB27 | -2.83 | 3.61 | 0.8600 | -14.08 | 8.429 |
|  | ABCAT11 | -10.85 | 3.61 | 0.0592 | -22.10 | 0.404 |
|  | PESAB20 | -1.270 | 0.658 | 0.1550 | -2.928 | 0.388 |
| PES | PESAB20 | 0.637 | 0.658 | 0.6040 | -1.021 | 2.295 |
