# Supplementary material for: Evidence for hybrid breakdown in production of red carotenoids in the marine invertebrate Tigriopus californicus
Source: PLoS One. 2021 Nov 8;16(11):e0259371. doi: 10.1371/journal.pone.0259371 (PMC8575244; doi:10.1371/journal.pone.0259371)
Supplement: S1 File — (DOCX) [file pone.0259371.s020.docx]

**1. Methods supplement**

**1.1 Experimental crosses and creation of recombinant inbred lines (RILs)**

We sampled from the following locations: La Bufadera (BUF; 31°72'37.25"N, 116° 72'18.15"W), San Diego (SD; 32°44'49.67"N, 117°15'13.34"W), Bird Rock (BR; 32°48'41.63"N, 117°15'58.11"W), Catalina Island (CAT; 33°23'57.45"N, 118°23'26.33"W), Abalone Cove (AB; 33°44'29.43"N, 118°22'45.59"W), Santa Cruz (SCN; 36°57'4.35"N, 122° 1'52.65"W), and Pescadero (PES; 37°15'43.79"N, 122°24'47.90"W). The experimental crosses used in this study were performed over several years at two different institutions. The creation of RILs and the reciprocal cross between SD and SCN were performed at the Scripps Institute of Oceanography at UC San Diego. The RILs from crossing SD and AB populations were created at Auburn University.

To create the early generation RILs from crossing SD and AB populations (completed in 2016), 20 males from SD and 20 virgin females from AB were paired together in petri dishes in triplicate in order to start with sufficient genetic variation to produce offspring with a variety of nuclear allelic combinations working against the single maternal mitochondrial type. When clasped pairs formed, they were moved to a well of a 6-well plate to produce F1 offspring. When F1 offspring began to form clasped pairs, these were separated and males from one of the three replicates were paired in a new well of another 6-well plate with females from one of the other cross replicates to avoid sibling pairings. When F2 offspring were formed, this process was repeated to produce the F3 generation. At the F3 generation we formed iso-female recombinant inbred lines by taking gravid F3 females and isolating them each in a well of a 6-well plate. When a female’s eggs hatched, the female was removed the offspring were allowed to inbreed to produce an F5 generation used for carotenoid analyses (see section below). We stopped hybrid generation at the 5^th^ generation as a precautionary measure to ensure enough lines could be sampled before dying out due to the effects of hybridization. This entire process was repeated by crossing males and females from the same population to make parental inbred lines (PILs).

Briefly, the multi-generational RILs from the crosses between (SDxBUF, SDxBR, ABxCAT, and ABxPES) were generated using the following methods. Males from one population were mated to virgin females of the second population in two replicates for the same reasons described above for the SDxAB cross. When these replicate pairings bore F1 offspring, males from one replicate were mated with females from the second replicate to avoid inbreeding. This process was repeated until the F3 generation. When F3 matings produced gravid females, the egg-bearing mothers were isolated in the well of a 6-well plate until their eggs hatched; once the eggs hatched, the females were removed. At this point, F4 siblings were allowed to inbreed, and mated pairs were again isolated to produce the F5 generation. This process was repeated until F7 offspring were produced, at which point F7 individuals were allowed to mate continuously to form recombinant inbred lines (originally formed from a single F3 mother) with overlapping generations. Discrete generations were maintained through the seventh generation in order to allow inbreeding among siblings and purge variation in alleles so that each line more closely represented a unique mitonuclear genotype after the effects of recombination in the first three non-inbred generations. These lines were maintained as continuous cultures prior to our study once the recombinatory genotypes were established so that logistically we could sample the lines as needed.

The reciprocal cross between SD and SCN populations was formed by pairing 40 males from SD with 40 virgin females from SCN and vice versa in two petri dishes. We started with 40 individuals per replicate instead of the 20 (described in the previous crosses) in order to minimize the chance of sibling pairings. Once every female in a dish formed eggs, males were removed, and the eggs allowed to hatch. When the F1 offspring began to form mating pairs, they were transferred to a new generation to produce F2 offspring. With this cross, we made no attempt to avoid sibling pairings in any generation. This process was repeated once more to produce F3 offspring which were then subjected to fitness and carotenoid measurements (described below). We did not form recombinant inbred lines with this cross due to time limitations imparted by the timing of the work amidst other projects and by the eventual onset of the SARS-CoV-2 pandemic in early 2020.

**1.2 Carotenoid bioconversion assays**

Prior to the start of the experiment, RIL copepods were switched to a diet of ground nutritional yeast until they became clear in color and deficient of both carotenoid precursors and the primary red carotenoid, astaxanthin [1, 2]. To test carotenoid bioconversion rate, carotenoid-deficient copepods were provided *Tetraselmis chuii* algae ad libitum for 7 days to mimic the copepods natural diet of photosynthetic algae rich with carotenoid precursors [3]. *Tetraselmis* *chuii* algae produces multiple dietary carotenoid precursors that can be converted into astaxanthin, most notably violaxanthin, lutein, and β-carotene [4]. Recent work shows that while *Tigriopus californicus* can convert violaxanthin and lutein into astaxanthin, the species does so only to a small degree and instead favors β-carotene as the substrate of choice to produce astaxanthin [5]. Thus, we assumed that the majority of free astaxanthin produced by copepods came primarily from the hydroxylation of β-carotene [5]. On the seventh day, copepods were moved to clean artificial saltwater for a minimum of 2 hours to clear any algae remaining in their gut. Copepods were then dried and weighed (±0.001 mg) before being stored in a microcentrifuge tube at -80°C until HPLC analysis (see below).

**1.3 Carotenoid extraction and HPLC analysis: SD and SCN cross**

Approximately 280mg of .5mm zirconia-silicate ruptor beads and 500uL of HPLC grade acetone was added to the microtubes containing dried and weighed copepods. Tubes were placed in a Mini-Beadbeater machine (Biospec Products) and homogenized at top speed for 30 seconds to break apart the copepod tissue and suspend the pigments in acetone. The acetone was then transferred into a 1.7mL microcentrifuge tube and spun down at 5000g for 5 minutes. The supernatant was then passed through a disposable 13mm .45um polypropylene filter (Whatman) with an additional 500uL of HPLC grade acetone into a fresh 1.7mL microcentrifuge tube. Tubes were evaporated to dryness in a centrivap concentrator (Labconco). The concentrated contents were resuspended in 50uL HPLC grade acetone, capped with nitrogen gas, and stored at -80°C until ready for HPLC pigment analysis.

**HPLC pigment analysis**

Samples were removed from the -80°C freezer. 40uL of HPLC grade water was added to each sample, and samples were vortexed and then spun down. The entire sample (90uL) was loaded into a 200uL flat bottom glass insert within 2mL autosampler vial (Agilent). Pigments were separated using an Agilent HPLC 1260 Infinity II LC system from a 75ul injection into a Waters Symmetry C8 column with a guard cartridge. We used mobile phases A) 50:25:25 Methanol: Acetonitrile: 0.25M Aqueous Pyridine and B) 20:60:20 Methanol: Acetonitrile: Acetone in a linear gradient as follows: starting at 100% A: 0% B to 60% A: 40% B over 18 min, then to 0% A: 100% B over 4 min, hold at 0% A: 100% B for 16 minutes, then back to 100% A: 0% B over 2 min and held for 8 minutes (Zapata, Rodríguez, & Garrido, 2000). Absorbance was measured at 450nm. Carotenoid peaks were identified by comparison to authentic standards (DHI) run through the same method. A final measurement of astaxanthin concentration was normalized to dry weight, resulting in a final measurement of micrograms of astaxanthin per milligram of copepod tissue.

**1.3 ATP synthesis and mitochondrial volume assays**

We measured ATP synthesis following an assay derived from Ellison & Burton, 2006. Ten males and ten females were homogenized with Dounce homogenizers. Their mitochondria were isolated by placing homogenized samples in a 4°C centrifuge at 1000g for 5 minutes. The supernatant was transferred into a new tube and placed in the centrifuge at 11000g for 10 minutes. The supernatant was discarded, and the pellet was resuspended in assay buffer (560mM sucrose, 100mM KCL, 10mM KH2PO4, and 70mM HEPES). Suspended mitochondria (25uL) from each sample were then added to 5uL of either Complex I (CI) substrate (100mM ADP, 800mM malate, 2M glutamate, and assay buffer) or Complex II (CII) substrate (100mM ADP, 1M succinate, 15uM rotenone, and assay buffer). Samples incubated for 10 minutes at 20°C on a thermo cycler. During incubation, ATP standards of 10,000nM, 5,000nM, 1,000nM, 500nM, 100nM, 100nM, 10nM, 5nM, and 0nM concentrations were added into an opaque white 96-well assay plate preloaded with 25uL of CellTitre-Glo (Promega). A time=0 control group was created, where 25uL of each mitochondrial resuspension was quickly added to 5uL of CI substrate, mixed briefly, and then immediately added into the assay plate preloaded with CellTitre-Glo. After incubation, 25uL of each sample was loaded into the plate and run immediately on a Fluoroskan Ascent FL plate reader (Thermo Labysystems). Using the generated standard curve and subtracting the control group, a final measurement of nanomoles of ATP produced per minute was reported.

The remainder (30uL) of each sample used in the ATP synthesis assay was saved for the citrate synthase assay [6]. Five microliters of each sample were added to 50uL of 200mM Tris buffer with 0.2% Triton-X (Sigma), 24uL DI water, 10uL 1mM DTNB (5, 5’-dithiobis(2-nitrobenzoic acid)), and 6uL acetyl coenzyme A (0.3mM). Absorbance was measured at 412nm for 5 minutes to check for background activity. Immediately after the background run, 5uL of 10mM oxaloacetic acid was added to each sample and read again at 412nm. The slope measured over 5 minutes was used to calculate the concentration of mitochondria in the original sample. ATP synthesis results were divided by this concentration to normalize the data.

**1.5 Statistical analyses**

All statistical analyses were performed in R [7] using the ‘lme4’ [8], ‘nlme’ [9], ‘lmerTest’ [10], ‘emmeans’ [11], ‘MASS’ [12], ‘agricolae’ [13], ‘tidyverse’ [14], and ‘MuMIn’ [15] packages. See R code in the supplement for notation about the role of each package. Figures were built in R using the ‘ggpubr’ [16] and ‘cowplot’ [17] packages.

We used mixed-effects linear models and pairwise contrasts corrected for multiple comparisons (using ‘emmeans’) to see if there were significant differences in astaxanthin accumulation among hybrid and non-hybrid copepod lines. When looking for differences in astaxanthin content between hybrid and non-hybrid copepods on average, we fit models that included a random effect of line ID to account for non-independence of data within each line. We also used linear models and paired contrasts corrected for multiple comparisons to look for statistically significant differences in ATP production and offspring development rate among hybrid and non-hybrid copepods lines.

Because we lacked data from female copepods with many of the crosses, we used male data to analyze the relationships between astaxanthin production, ATP, mitochondrial volume, and offspring development time among all of the lines. We used linear models to assess the relationship between astaxanthin, ATP production, mitochondrial volume, and offspring development rate. We included mitochondrial type (i.e., the mito type of the maternal population in hybrid crosses) as a random effect. Before fitting the models, ATP, mitochondrial volume, and development time data were log transformed, scaled, and centered to achieve normality (assessed by inspecting model residuals). We averaged replicates from each line to avoid pseudoreplicaiton.

When assessing if there were significant differences in astaxanthin production, ATP production and offspring development rate between generations from the reciprocal cross between SD and SCN populations, we used a linear model and pairwise contrasts corrected for multiple comparisons including a random effect of maternal mito type to account for non-independence of data from each line. To examine the relationship between astaxanthin production, ATP, mitochondrial volume, and offspring development rate with the reciprocal cross, we averaged replicates from each line to avoid pseudoreplicaiton and used linear models. However, because we had a large number of replicates per generation (compared to ATP and mitochondrial volume measurements) when assessing offspring development rate, we also analyzed the relationship between offspring development rate and astaxanthin production within each generation using separate linear models.

**1.6 Phylogenetic analysis**

The evolutionary history of the *Tigriopus californicus* populations used in this study was inferred by using the Maximum Likelihood method and Tamura-Nei model, using *Tigriopus japonicus* as a rooted outgroup [18]. The tree with the highest log likelihood (-3168.00) is shown. The percentage of trees in which the associated taxa clustered together is shown next to the branches. Initial tree(s) for the heuristic search were obtained automatically by applying Neighbor-Join and BioNJ algorithms to a matrix of pairwise distances estimated using the Tamura-Nei model, and then selecting the topology with superior log likelihood value. The tree is drawn to scale, with branch lengths measured in the number of substitutions per site. This analysis involved 8 nucleotide sequences of the CO1 gene. There were a total of 613 positions in the final dataset. Evolutionary analyses were conducted in MEGA X [19]. Gene sequences were generously shared with us by Reggie Blackwell.

**References**

1. Powers MJ, Hill GE, Weaver RJ. An experimental test of mate choice for red carotenoid coloration in the marine copepod Tigriopus californicus. Ethology. 2020;126(3):344-52.

2. Weaver RJ, Cobine PA, Hill GE. On the bioconversion of dietary carotenoids to astaxanthin in the marine copepod, Tigriopus californicus. Journal of Plankton Research. 2018;40(2):142-50.

3. Vittor BA. Effects of the environment on fitness related life history characters in Tigriopus californicus: Thesis (Ph. D.)--Oregon, Dept. of Biology; 1971.

4. Ahmed F, Fanning K, Netzel M, Turner W, Li Y, Schenk PM. Profiling of carotenoids and antioxidant capacity of microalgae from subtropical coastal and brackish waters. Food Chem. 2014;165:300-6. Epub 2014/07/21. doi: 10.1016/j.foodchem.2014.05.107. PubMed PMID: 25038679.

5. Prado-Cabrero A, Saefurahman G, Nolan JM. Stereochemistry of Astaxanthin Biosynthesis in the Marine Harpacticoid Copepod Tigriopus Californicus. Mar Drugs. 2020;18(10). Epub 2020/10/09. doi: 10.3390/md18100506. PubMed PMID: 33028032; PubMed Central PMCID: PMCPMC7600253.

6. Spinazzi M, Casarin A, Pertegato V, Salviati L, Angelini C. Assessment of mitochondrial respiratory chain enzymatic activities on tissues and cultured cells. Nature protocols. 2012;7(6):1235-46.

7. R_Core_Team. R: A language and environment for statistical computing. 3.6.0 ed: Vienna, Austria; 2019.

8. Bates D, Mächler M, Bolker B, Walker S. Fitting linear mixed-effects models using lme4. arXiv preprint arXiv:14065823. 2014.

9. Pinheiro J, Bates D, DebRoy S, Sarkar D, Team RC. nlme: Linear and nonlinear mixed effects models. R package version. 2013;3(1):111.

10. Kuznetsova A, Brockhoff PB, Christensen RH. lmerTest package: tests in linear mixed effects models. Journal of statistical software. 2017;82(13):1-26.

11. Lenth R, Singmann H, Love J, Buerkner P, Herve M. Emmeans: Estimated marginal means, aka least-squares means. R package version. 2018;1(1):3.

12. Venables WN, Ripley BD. Modern applied statistics with S-PLUS: Springer Science & Business Media; 2013.

13. De Mendiburu F. Agricolae: statistical procedures for agricultural research. R package version. 2014;1(1).

14. Wickham H. Tidyverse: Easily install and load the ‘tidyverse’. R package version. 2017;1(1):2017.

15. Barton K. MuMIn: multi-model inference. <http://r-forge> r-project org/projects/mumin/. 2009.

16. Kassambara A. ggpubr:“ggplot2” based publication ready plots. R package version 01. 2018;7.

17. Wilke CO. cowplot: streamlined plot theme and plot annotations for ‘ggplot2’. CRAN Repos. 2016;2:R2.

18. Tamura K, Nei M. Estimation of the number of nucleotide substitutions in the control region of mitochondrial DNA in humans and chimpanzees. Molecular biology and evolution. 1993;10(3):512-26.

19. Kumar S, Stecher G, Li M, Knyaz C, Tamura K. MEGA X: molecular evolutionary genetics analysis across computing platforms. Molecular biology and evolution. 2018;35(6):1547-9.
