# Supplementary material for: Evidence for hybrid breakdown in production of red carotenoids in the marine invertebrate Tigriopus californicus
Source: PLoS One. 2021 Nov 8;16(11):e0259371. doi: 10.1371/journal.pone.0259371 (PMC8575244; doi:10.1371/journal.pone.0259371)
Supplement: Attachment — Submitted filename: Response to Reviewers.docx [file pone.0259371.s023.docx]

**For ease of review, we have reproduced the reviewer comments below. Our responses to individual comments follow in blue text.**

**Reviewer #1:**

Reviewer 1, thank you for the constructive feedback. We agree with your point that the data from our study are complex, and conclusions are not as clear as we would have initially hoped when designing the experiments. However, we have worked to address your questions, provide additional background information, and add further clarification to our results. We believe the manuscript is improved with these additions being guided by your comments.

The authors investigate whether production of the red keto-carotenoid astaxanthin from dietary precursors is disrupted in inbred hybrid lines (RIL’s) generated from crosses between populations of the marine copepod Tigriopus californicus in which incompatibilities between nuclear and mitochondrial genes involved in the process could be exposed. Production of astaxanthin is an enzymatic process that has been hypothesized to signal individual condition in some organisms, possibly through shared metabolic pathways involved in oxidative phosphorylation.

The authors do observe reduced capabilities to produce astaxanthin in males (why not females?!) of some of the created inbred lines compared to the parental forms that may be attributed to an effect of the genetic background, but little in the way of a predicted link to mitochondrial performance. Also, the results are muddied by an unexpected effect of diet (yeast-based without carotenoids vs algae-based) used to propagate the lines, such that some of the results, e.g., how astaxanthin production is related to ATP production, went in opposite directions between lines grown on the two different diets.

The lack of a clear effect on carotenoid bioconversion in females, but not males, is interesting. On lines 436-439 of the original submitted text, we did previously include some speculation on this matter. We referred to a handful of studies that indicated female copepods may be generally more resistant to external (and possibly internal) perturbations. Males specifically may show greater variation in respiratory traits (reference 64 in original submission).

64. Li N, Flanagan BA, Partridge M, Huang EJ, Edmands S. Sex differences in early transcriptomic responses to oxidative stress in the copepod Tigriopus californicus. BMC genomics. 2020;21(1):1-11.

In terms of astaxanthin specifically, female *T. californicus* must sequester enough astaxanthin not only for themselves, but also for their developing offspring. Carotenoids must be transferred to the developing eggs in order to provide freshly hatched offspring with the UV-protection they need to survive their first two naupliar instars. During this early period, offspring (which are the same bright red as adults) cannot eat and, therefore, cannot acquire their own carotenoid precursors. We have added this extra bit of speculation nearly verbatim to the tail end of the lines mentioned above (lines 477-483 in the resubmission).

Personally, I feel that the study raises more questions than it answers. Fortunately, providing a bit more background information on the system studied may alleviate some of these deficiencies.

How many chromosomes or linkage groups does Tigriopus have? Of course, it would have been interesting to identify which chromosomes or linkage groups are associated with the inbred lines with reduced pigmentation, but I can accept that this is a different study. What could have been done to prevent RIL’s with specific combinations of nuclear/mitochondrial genes resulting in low viability to be selected against or to go “extinct”? Also, it is unfortunate that the authors weren’t able to use the same inbred lines to compare the results for the two diets.

*T. californicus* has a fully sequenced genome with 12 chromosomes (see Barreto et al. 2018).  We agree it would be informative to see which linkage groups are associated with reduced or increased carotenoid bioconversion. However, such a study on a quantitative trait would require whole genome sequencing of a large number of inbred lines to determine which genomic regions are consistently associated with coloration rather than just those diverged due to drift. As alluded to in the above reviewer comment, this work would be beyond the scope of this study.

Barreto FS, Watson ET, Lima TG, Willett CS, Edmands S, Li W, et al. Genomic signatures of mitonuclear coevolution across populations of Tigriopus californicus. Nat Ecol Evol. 2018;2(8):1250-7. Epub 2018/07/11. doi: 10.1038/s41559-018-0588-1. PubMed PMID: 29988158.

We also agree that it would have been idea to have a more complete representation of inbred lines across both diets. Ideally, it would be most advantageous to have many more inbred lines from each of the experimental crosses represented in the present study. Unfortunately, the study was limited by the availability of viable crosses during the window of time in which the study was conducted (the facility shutdowns across the United States during the SARS-CoV-2 pandemic did no favors in this regard). At this time, it is unclear if there is any way to “rescue” RIL’s with severely incompatible mitonuclear combinations that are in danger of extinction. Experiments have shown that backcrossing to the maternal population can reintroduce compatible nuclear alleles and rescue some viability. However, there have also been experiments that show that mitonuclear incompatibilities differ among each cross (Lima et al. 2019; Healy and Burton 2020, Pereira et al. 2021). Therefore, “fixes” for dying lines may not work equally among crosses. Moreover, fixes that alter the environment of the crosses to relieve the effects of mitonuclear incompatibilities may only make it more difficult to compare fitness measures among lines.

We have added a bit of this clarification and information (lines 450-458 of the resubmission) to the start of the discussion of the manuscript in hopes that it provides readers some background on the nuances of inbred lines with quantifiable mitonuclear dysfunction.

The authors do come up with reasonable explanations for their results, including those linked to diet, but without additional measures of mitochondrial function to constrain them, it is hard to judge whether they are correct. However, on whole I feel that the authors do provide a fairly balanced treatment of their findings, even if they are somewhat inconclusive. They certainly raise questions that could, and should, be addressed in future work, building on the present work.

Thank you. We agree that additional context beyond ATP production is warranted to both better contextualize mitochondrial function and to explore potential relationships with the redox-reaction-driven carotenoid bioconversion processes in *Tigriopus* copepods. Indeed, starting with ATP is only perhaps the simplest method of quantifying mitochondrial dysfunction; it cannot fully encapsulate dysfunction without further context. For a future study, we are in the process of inducing specific dysfunction in the mitochondria of *T. californicus* copepods using chemical inhibitors (similar to the work of Cantarero et al. 2017, 2020), and we will be measuring additional mitochondrial-based traits such as oxygen consumption and electron donor/acceptor ratios. This should provide more context to mitochondrial efficiency alongside carotenoid bioconversion.

Cantarero A, Alonso-Alvarez C. Mitochondria-targeted molecules determine the redness of the zebra finch bill. Biol Lett. 2017;13(10). Epub 2017/10/27. doi: 10.1098/rsbl.2017.0455. PubMed PMID: 29070589; PubMed Central PMCID: PMCPMC5665772.

Cantarero A, Mateo R, Camarero PR, Alonso D, Fernandez-Eslava B, Alonso-Alvarez C. Testing the shared-pathway hypothesis in the carotenoid-based coloration of red crossbills. Evolution. 2020;74(10):2348-64. Epub 2020/08/05. doi: 10.1111/evo.14073. PubMed PMID: 32749066.

Specific (very minor) comments:
Line 84: “crossbills” (no hyphenation). Fixed
Line 105: remove extra space between “population” and “[37, 41]”. Fixed
Line 113: Should “complexes” be pluralized? Fixed
Line 160: “established” Fixed
Line 200: a description of, at the very least, the mobile phase would be appreciated. This has been added to this line (lines 209-213 of resubmission).
Between lines 244 and 257 μL (not uL). Fixed
Line 250: “Spinazzi, Casarin (52)” shouldn’t this be “Spinazzi et al. [52]”? Correct. The right citation has been added.
Line 282: I am not sure that the word “Recombinant” is the best (or even correct) term here. It conjures images of recombinant DNA, which involves a different process. But I understand what you mean.

The term recombinant is the correct term. The term “recombinant” in “recombinant inbred lines” does, in fact, refer to recombinant DNA. This descriptor has been used to describe the process of hybrid generation used for decades with *Tigriopus californicus* (for example, summarized in Burton et al. 2006; Burton et al. 2013; and Barreto et al. 2018). The name is so chosen as to encapsulate the process by which hybrid crosses are formed, wherein F1, F2, and F3 hybrids are formed with a no-inbreeding strategy, creating many different combinations of nuclear alleles from the parental populations. Offspring of F3 mothers are then used to create inbred lines (the RILs), capturing a unique combination of nuclear alleles in each line. This process is described in the supplemental methods file included in the original submission. Since we used RILs in this study, we need to make sure we keep the nomenclature the same to connect to decades of past hybrid studies in *T. californicus*.

Burton R, Ellison C, Harrison J. The sorry state of F2 hybrids: consequences of rapid mitochondrial DNA evolution in allopatric populations. The American Naturalist.

Barreto FS, Watson ET, Lima TG, Willett CS, Edmands S, Li W, et al. Genomic signatures of mitonuclear coevolution across populations of Tigriopus californicus. Nat Ecol Evol. 2018;2(8):1250-7. Epub 2018/07/11. doi: 10.1038/s41559-018-0588-1. PubMed PMID: 29988158.

Burton RS, Pereira RJ, Barreto FS. Cytonuclear genomic interactions and hybrid breakdown. Annual Review of Ecology, Evolution, and Systematics. 2013;44:281-302

Line 307-308: “hybrid breakdown” may be too strong a word here. Replaced “hybrid breakdown” with the less-specific word “difference”
Line 405: remove period before “T. californicus“ Fixed
Line 492: remove space before “[76]” Fixed
Line 699: 59 is out of place The “59” is a part of the citation #55 (Parry et al. 2020). The PLOS ONE citation style in EndNote removes the 3 from 359 so that the page numbers of the citation read “349-59”

**Reviewer #2:**

Reviewer 2, thank you for your suggestions and feedback. Your comments on our manuscript were very helpful. We have worked to address your concerns by including the extra data analyses that you mentioned in your first major comment and by adding the needed information you identified in your other comments. We think these changes have improved the manuscript and we hope that we have adequately addressed your concerns. Please see below for our responses to your individual comments.

The authors present a set of experiments looking at relationships between astaxanthin concentration, mitochondrial function, and fitness in copepods. The authors hypothesized that astaxanthin would be correlated with mitochondrial function. They also evaluated links between mitochondrial function and fitness. The data collected revealed some unexpected and interesting relationships between these three variables that will make a valuable contribution to the field. Overall, I feel that this manuscript is well-written and addresses a gap in the literature of linking fitness to these other two variables that have been studied previously (astaxanthin, mitochondrial function) in this system. I also think that the study design and data analyses are mostly appropriate for addressing the hypotheses (see the one exception in my notes).

There are multiple aspects of this manuscript that I liked including:
• Fitness is rarely measured in this context, and copepods are a great system for it, so that is a much appreciated contribution.
• I appreciate the inclusion of R code for reproducibility of data analysis.
• I also appreciate the honesty of results presentation by publishing all of the results, not just the second experiment, after the authors realized that the diet might have a confounding effect. This helps those working in the system to avoid the same mistake.

Thank you very much!

However, there are a few aspects that need additional attention or clarification:
1. The authors mentioned “efficiency of carotenoid ketolation” in their hypothesis (line 118), however, astaxanthin concentration is not necessarily a measure of this, because it ignores variation in dietary carotenoids (e.g. beta-carotene) that were not converted into astaxanthin. If dietary carotenoids were measured (as I assume they were since beta-carotene and other dietary carotenoid standard curves were mentioned in the methods), then they should be used to control for differences in absorption of dietary carotenoids.

Thank you. This is a good point. We have now included a comparison of the detectable dietary carotenoids across our inbred lines (β-carotene and hydroxyechinenone). There was considerable overlap in the concentration of dietary carotenoids found across the RILs, and hybrid and parental lines did not significantly differ from one another in any meaningful way (supplemental figure 9; new figure).

There are many ways this could be addressed by the authors that would be satisfying. Some options include: 1) providing some evidence that total carotenoids (dietary + astaxanthin) did not vary substantially in your sample, 2) using total carotenoids and/or dietary carotenoids to make a ratio (e.g. astaxanthin : beta-carotene) that more accurately reflects bioconversion (not just concentration of astaxanthin), or 3) including total carotenoids in the models. If the authors don’t have data on dietary carotenoids in the sample for whatever reason, then a citation on prior work that has looked at dietary vs. astaxanthin concentrations in copepods would be acceptable.

This is an excellent point. Based on this feedback, we included extra data and analyses using the ratio of astaxanthin to dietary carotenoids (option 2 in your comment above) in the manuscript alongside our original astaxanthin data. Unfortunately, we did not perform the necessary saponification steps during carotenoid extractions to quantify esterified carotenoids so total carotenoid content was unavailable to us. We present this new data and analyses on the yeast-fed RILs and PILs (the crosses between SDxBUF, SDxBR, ABxCAT, and ABxPES; originally the main data showed in Figure 2 of the original submission). We had no further HPLC data on the cross between SDxAB. As noted in our original submission (lines 207-214), the samples from the cross between SDxSCN (the lines switched to algae during fitness and mito measures) were analyzed separately from the rest of our samples using a different HPLC method. This method did not detect appreciable concentrations of dietary carotenoids from most of the SDxSCN samples. In a few samples, echinenenone/hydroxyechinenone (putative) and lutein (not used to make astaxanthin by *T. californicus*) were observed (see data file in “S3_File_Hybrid_carot_master_data_workbook”, sheet named “All carots identified in SDxSCN”). This datasheet contains the carotenoid values recorded for this cross, and a few others from other experiments that were run concurrently. Therefore, we were not able to include updated analyses using the ratio of astaxanthin to dietary carotenoids for the SDxSCN cross. We hope that the added analyses using the ratio of carotenoids in the other RILs will be sufficient.

We would like to note here that prior work has shown that the primary carotenoid detected in *T. californicus* copepods is free astaxanthin (Weaver et al. 2018; Prado-Cabrero et al. 2021). This also seems to be the case in our copepods (mean ug mg^-1^ carotenoid across all samples ± st. dev; β-carotene: 0.3 ± 0.3, hydroxyechinenone: 0.04 ± 0.02, astaxanthin: 0.25 ± 0.11). Therefore, astaxanthin content on its own is likely to reflect the majority of variation in carotenoid pigmentation across copepods. We have added this extra bit of background information to lines 100-103 of the resubmitted manuscript.

We found that the ratio of astaxanthin to dietary carotenoids reflected mostly the same pattern across our inbred lines that we observed with astaxanthin alone. Interestingly, we found that when we contrasted the carotenoid ratios with Complex I ATP production, the relationship was not as strong, but was still negative. When we contrasted the carotenoid ratios with offspring development time, we found a significant, positive relationship (as compared to no clear relationship between development time and astaxanthin). There was little by way of differences across the lines in dietary carotenoid concentrations (new S9_Fig).

We have updated the results and discussion to show and consider these analyses (lines 338-345, 354-358, 385-388, 499-501, 547-550). We have also expanded our supplemental figures with five new figures (now Figures S6-S10) to show the new set of results. Truth told; we are not sure that the new analyses offer much by way of making conclusions clearer. As noted by reviewer 1, there are many new questions raised by this study. However, we think the added data certainly makes for a more interesting dataset that should improve the impact of the study if published.

2. In general, the hybrid-making methods (experimental crosses and creation of recombinant inbred lines) are lacking sufficient justification, either in the form of citations or written explanation by the authors’ experiences. Some justification could address why specific numbers of generations were used to create the hybrids, why discrete generations were maintained until the seventh generation when offspring were allowed to mate continuously, why with a particular cross (SD x AB) astaxanthin bioconversion was only assayed at the 5th generation, etc. There are multiple instances like this where the how is thoroughly described but not the why. At least some should be in the main text, but most of this justification could go in the supplemental materials.

We agree that the methods could use further explanations and citations. We have added further clarification to these methods in the main text on lines 167-170 and in the supplemental methods section 1.1. We added much of the extra details to the supplemental methods to preserve readability of the main text.

3. There are question marks like this “(???)” in the HPLC pigment analysis section of the supplemental files that should be removed. Fixed.

4. In the methods, the authors mention that gravid females were fed powdered Spirulina instead of yeast. Is this the same algae diet that is mentioned throughout the manuscript or is this a different algae diet? This should be explicitly noted either way, since it is the first mention of the word Spirulina (the rest of the manuscript just refers to algae diet). If it is a different diet, then the nutritional differences between it and the other algae diet should be described and justified.

We have added clarification to the methods on this point. As noted in the new lines added to this section (lines 229-233 of resubmission), Spirulina is a powdered algae mainly consisting of cyanobacteria/blue-green algae. Its main pigment content is anthocyanins, rather than carotenoids.

5. In the statistics section of methods (line 264), the authors describe “astaxanthin accumulation” which could be represented by multiple possible data types (e.g. amount, concentration, proportion). I didn’t see that it meant concentration specifically until I looked at Figure 2 and saw that the y-axis was concentration (ug/mg). The text should reference this more explicitly (that accumulation refers to concentration of astaxanthin in this study).

We have worked to specify that our measured response variable was carotenoid concentration across the manuscript where it applies to the analyses specifically. We started by changing the word ‘accumulation’ to ‘concentration’ in the lines that you referred to in your comment. The other relevant areas where we made sure to change this are in the figure descriptions.

**Comments from Journal:**

1. Please ensure that your manuscript meets PLOS ONE's style requirements, including those for file naming.

We ensured that our reference manager, EndNote, was using the latest PLOS style guide file downloaded directly from the journal website. We believe we have the figure and supplemental file naming conventions correct as well based on our review of the format guide pdf documents. We’ve also checked our figures in the online PACE tool and it identified no problems.

2. In your Methods section, please provide additional information regarding the permits you obtained for the collection of copepod samples. Please ensure you have included the full name of the authority that approved the field site access and, if no permits were required, a brief statement explaining why.

This has been added on lines 132-133.

**Author-elected changes:**

We would also like to note that in our resubmission, we have edited the first two pages of the introduction to improve readability. The ideas presented and literature cited are the same (with the exception of a few added citations), but the technical details of carotenoid biology are simplified, reordered, or cleaned up to help the reader understand the relevant background.
